# Supplementary material for: Coronatine-Based Gene Expression Changes Impart Partial Resistance to Fall Armyworm (Spodoptera frugiperda) in Seedling Maize
Source: Genes (Basel). 2023 Mar 16;14(3):735. doi: 10.3390/genes14030735 (PMC10048583; doi:10.3390/genes14030735)
Supplement: Supplementary file 1 [file genes-14-00735-s001.zip › Supplemental Data.pdf]

Coronatine based gene expression changes impart partial resistance to fall armyworm (*Spodoptera frugiperda*) in maize seedling.

Yuxuan Lou[1], Xiaoxiao Jin[1], Zhiguo Jia[1], Yuqi Sun[1], Yiming Xu[1], Zihan Liu[1], Shuqian Tan[2*], Fei Yi[1*], Liusheng Duan[3]

1 Engineering Research Center of Plant Growth Regulator, Ministry of Education & College of Agronomy and Biotechnology, China Agricultural University, Beijing 100193, China.

2 MOA Key Lab of Pest Monitoring and Green Management, Department of Entomology, College of Plant Protection, China Agricultural University, Beijing 100193, China.

3 Beijing University of Agriculture, Beijing 100193, China.

*Correspondence: yifei56@cau.edu.cn (F.Y.); SQTan@cau.edu.cn(S.T.)

Keywords: coronatine; maize; *Spodoptera frugiperda*; RNA-seq; insect resistance mechanism

Supplemental Figures:

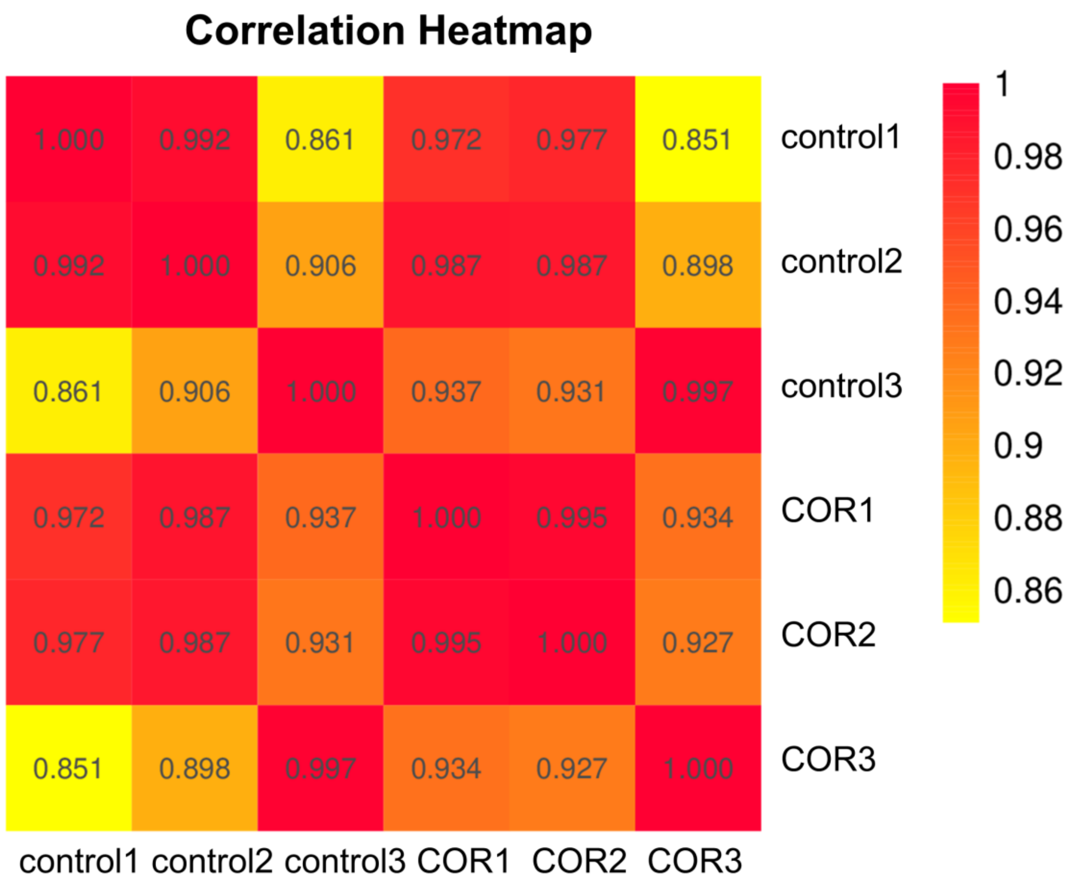

Supplemental Figure S1: The correlation heatmap of six samples.

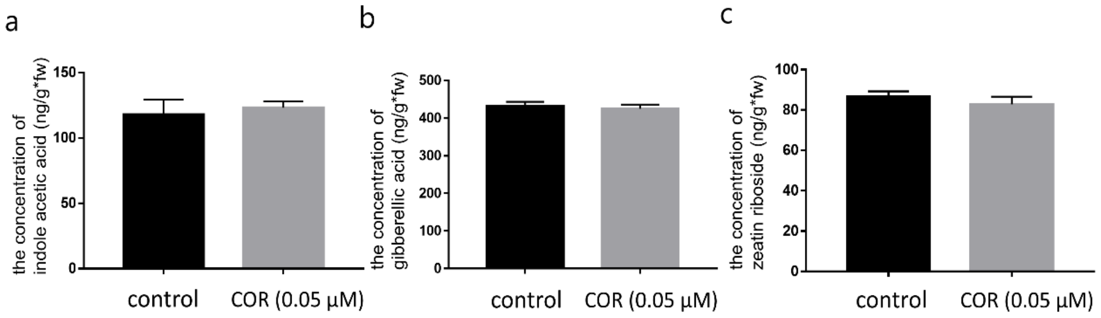

Supplemental Figure S2: Three plant hormones whose contents did not change after treatment with COR.

(a), (b) and (c): Changes of IAA (a), GA (b) and ZR (c) contents in maize leaves induced by 0.05 μM COR. The data were presented as means ± SE (n = 3) and analyzed by Student's t-test. SE is represented by error bars. * Indicates that the difference is significant, and the significant degree α is 0.05.

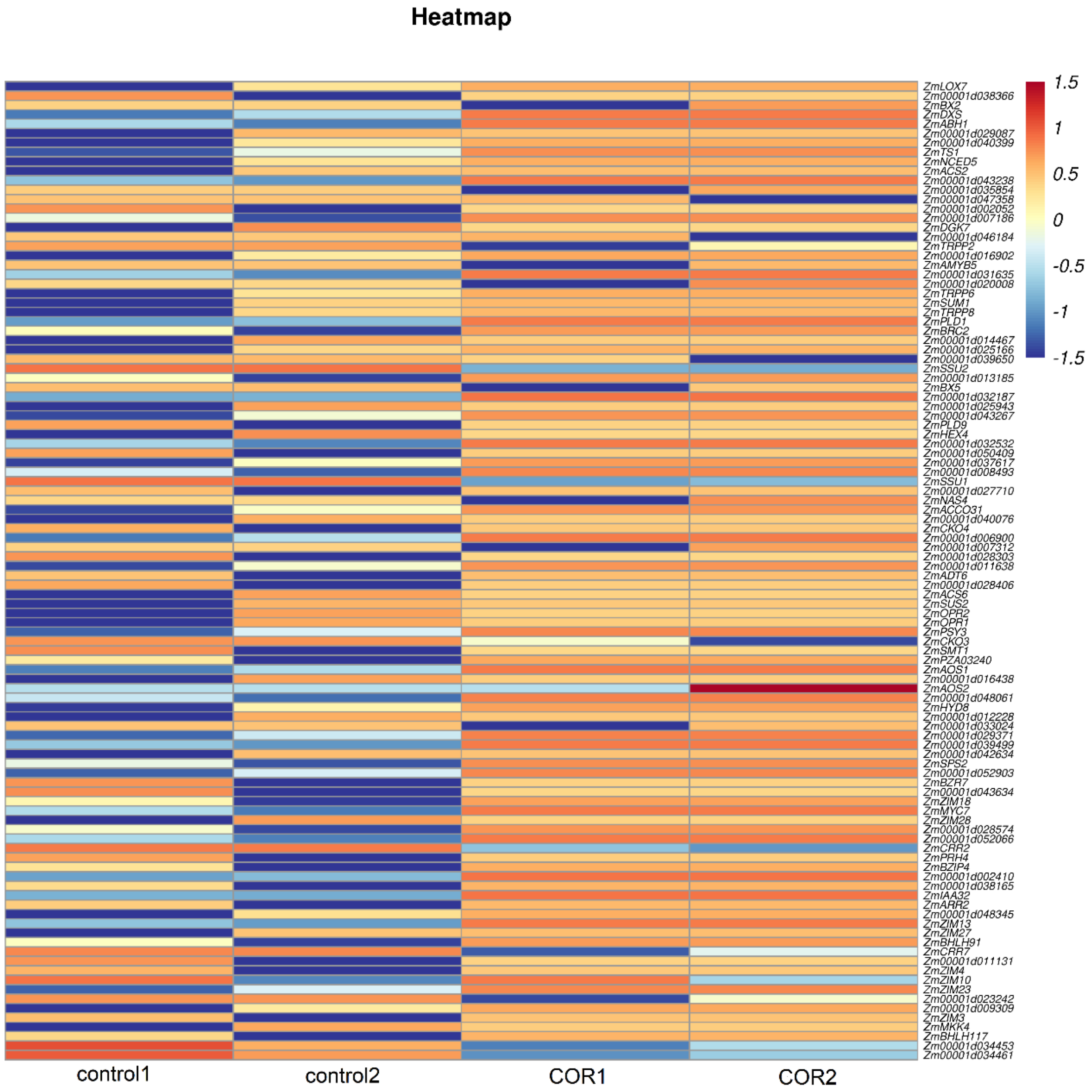

Supplemental Figure S3: The expression of genes related to biosynthesis of secondary metabolites, benzoxazinoid biosynthesis and plant hormone signal transduction in KEGG database.

The data were normalized by log2 and Z-score, the closer to 1.5, the higher the relative gene expression, and the closer to -1.5, the lower the relative gene expression.

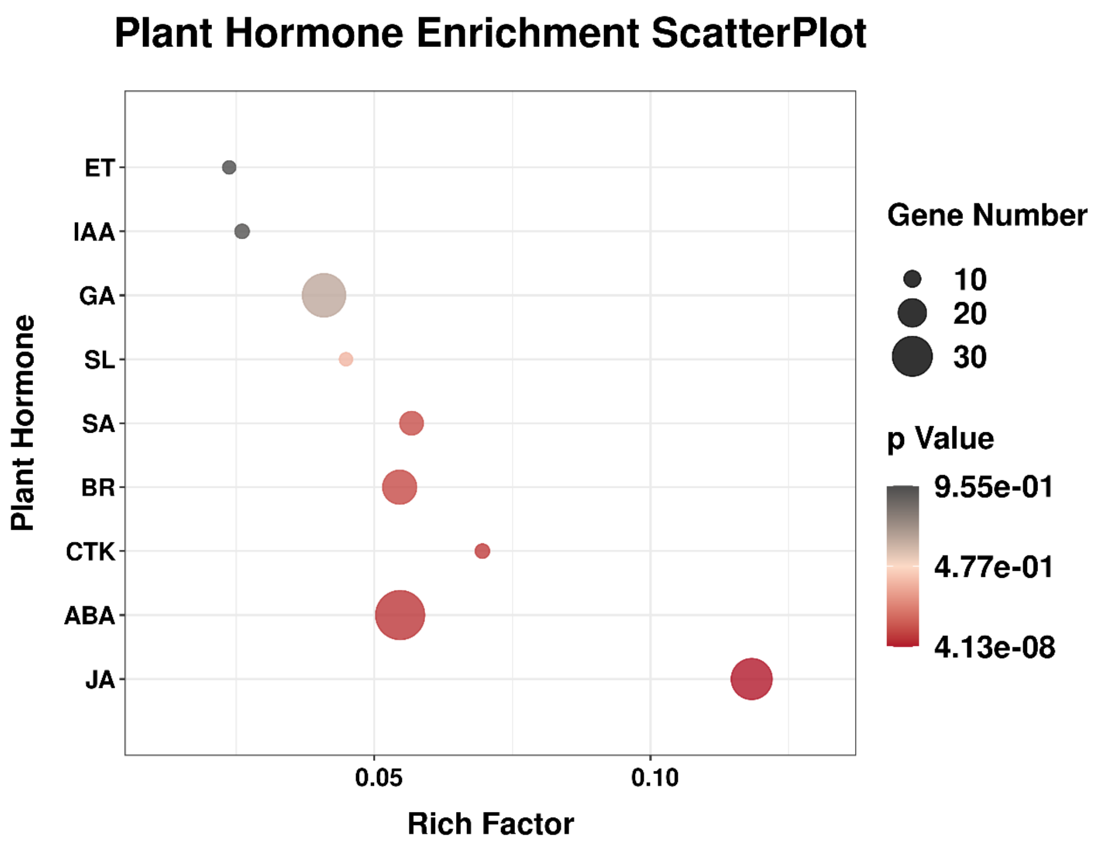

Supplemental Figure S4: Enrichment analysis of genes related to plant hormones.

Supplemental Tables:

Supplemental Table S1. GenBank ID of genes related to benzoxazine biosynthesis.

Gene symbol	Full name	gene ID_V4
ZmBX1	benzoxazinone synthesis 1	Zm00001d048709
ZmBX2	benzoxazinone synthesis 2	Zm00001d048710
ZmBX3	benzoxazinone synthesis 3	Zm00001d048702
ZmBX4	benzoxazinone synthesis 4	Zm00001d048703
ZmBX5	benzoxazinone synthesis 5	Zm00001d048705
ZmBX6	benzoxazinone synthesis 6	Zm00001d048634

Supplemental Table S2. Differentially expressed genes related to plant hormone

Gene ID	Gene symbol	Module	Hormone_type
Zm00001d002540	cdpk5	GGPPS	GA_Biosynthesis
Zm00001d003533	ts1(lox8)	PIFs	GA_Signal_Transduction
Zm00001d004664	cyp27	bZIP	GA_Signal_Transduction
Zm00001d005164	cdpk33	NAC	GA_Signal_Transduction
Zm00001d005208	nactf5	bHLH	GA_Signal_Transduction
Zm00001d005813	zim13	GAMYB	GA_Signal_Transduction
Zm00001d005951	hb54	GRAS	GA_Signal_Transduction
Zm00001d005969		bHLH	GA_Signal_Transduction
Zm00001d006065		bHLH	GA_Signal_Transduction
Zm00001d006551	gras49	bZIP	GA_Signal_Transduction
Zm00001d006621	cdpk32	GGPPS	GA_Biosynthesis
Zm00001d006701	gras75	GID1	GA_Signal_Transduction
Zm00001d008307	nactf97	bHLH	GA_Signal_Transduction

Zm00001d008308	nactf97	bHLH	GA_Signal_Transduction
Zm00001d011131	prh18	NAC	GA_Signal_Transduction
Zm00001d011132	prh19	NAC	GA_Signal_Transduction
Zm00001d011614	myb24	NAC	GA_Signal_Transduction
Zm00001d011666	cdpk21	bHLH	GA_Signal_Transduction
Zm00001d011890	cko3	GA2ox	GA_Biosynthesis
Zm00001d012304		bZIP	GA_Signal_Transduction
Zm00001d012401	prh4	GID1	GA_Signal_Transduction
Zm00001d012527	nactf23	NAC	GA_Signal_Transduction
Zm00001d012642		bHLH	GA_Signal_Transduction
Zm00001d013003	nactf6	NAC	GA_Signal_Transduction
Zm00001d013098	ao2	GRAS	GA_Signal_Transduction
Zm00001d013109	cdpk1	GRAS	GA_Signal_Transduction
Zm00001d013185	aos5	NAC	GA_Signal_Transduction
Zm00001d013220		bZIP	GA_Signal_Transduction

Zm00001d014253	zim1	bHLH	GA_Signal_Transduction
Zm00001d014658	mpk5	GRAS	GA_Signal_Transduction
Zm00001d015100	cdpk22	bZIP	GA_Signal_Transduction
Zm00001d015515	wrky48	NAC	GA_Signal_Transduction
Zm00001d015614	myb5	bZIP	GA_Signal_Transduction
Zm00001d017422	hb41	bZIP	GA_Signal_Transduction
Zm00001d017762	abh1	NAC	GA_Signal_Transduction
Zm00001d018178	bzip4	GA2ox	GA_Biosynthesis
Zm00001d018799	cipk23	bZIP	GA_Signal_Transduction
Zm00001d018819	nced5	bHLH	GA_Signal_Transduction
Zm00001d018973	iaa32	bZIP	GA_Signal_Transduction
Zm00001d019060	dxs2	NAC	GA_Signal_Transduction
Zm00001d019207	nactf4	GRAS	GA_Signal_Transduction
Zm00001d019692	zim38	bHLH	GA_Signal_Transduction
Zm00001d020008	igps1	bHLH	GA_Signal_Transduction

Zm00001d020614	zim28	bHLH	GA_Signal_Transduction
Zm00001d021236	ocl3	SAUR	IAA_Signal_Transduction
Zm00001d021817		ARF	IAA_Signal_Transduction
Zm00001d021835	cdpk19	NITs	IAA_Biosynthesis
Zm00001d021946	gras38	YUCCA	IAA_Biosynthesis
Zm00001d022139	zim23	TSB1	IAA_Biosynthesis
Zm00001d024522		YUCCA	IAA_Biosynthesis
Zm00001d025141		ABCB	IAA_Transportation
Zm00001d025524	lox7	CUL	IAA_Signal_Transduction
Zm00001d025752		AUX/IAA	IAA_Signal_Transduction
Zm00001d025903		AUX/IAA	IAA_Signal_Transduction
Zm00001d026269		SAUR	IAA_Signal_Transduction
Zm00001d026594	crr2	SAUR	IAA_Signal_Transduction
Zm00001d027443	hyd6	SAUR	IAA_Signal_Transduction
Zm00001d027585	cki3	SAUR	IAA_Signal_Transduction

Zm00001d027900	zim27	ERF1	JA_Signal_Transduction
Zm00001d028282	aos2	JAZ	JA_Signal_Transduction
Zm00001d028574		KEG	JA_Signal_Transduction
Zm00001d028742	fad8	AOS(alleneoxidesynthase)	JA_Biosynthesis
Zm00001d029684	pld9	MYC2	JA_Signal_Transduction
Zm00001d030028		JAZ	JA_Signal_Transduction
Zm00001d031725	tcptf21	OPR(12-Oxo-PDAReductase)	JA_Biosynthesis
Zm00001d033049	zim3	PLD	JA_Biosynthesis
Zm00001d033050	zim18	LOX(linolenatelipoxygenase)	JA_Biosynthesis
Zm00001d033624	lox4	ORA59	JA_Signal_Transduction
Zm00001d033848		YAB	JA_Signal_Transduction
Zm00001d033862	acs6	YAB	JA_Signal_Transduction
Zm00001d033957		HAC1	JA_Signal_Transduction
Zm00001d034109		HDA19	JA_Signal_Transduction
Zm00001d034453		JAZ	JA_Signal_Transduction

Zm00001d034461	tsah1	JAZ	JA_Signal_Transduction
Zm00001d035440	bzip12	PP2C	ABA_Signal_Transduction
Zm00001d037221	tcptf1	PP2C	ABA_Signal_Transduction
Zm00001d038165	GID1	SUC5	ABA_Signal_Transduction
Zm00001d038340		RGLG1/RGLG5	ABA_Signal_Transduction
Zm00001d039037		CAMTA	ABA_Signal_Transduction
Zm00001d039670	pld1	CIPK	ABA_Signal_Transduction
Zm00001d040318	nactf66	SnRK2	ABA_Signal_Transduction
Zm00001d041920	bzip8	MAPK	ABA_Signal_Transduction
Zm00001d041996		AKT(7 和 8 是 KAT1)	ABA_Signal_Transduction
Zm00001d042267	arftf1	SnRK2	ABA_Signal_Transduction
Zm00001d042313	camta3	RboH	ABA_Signal_Transduction
Zm00001d043062	wrky4	ABRE	ABA_Signal_Transduction
Zm00001d043293	cko4	ATHB	ABA_Signal_Transduction
Zm00001d043706		SAUL1/PUB44	ABA_Biosynthesis

Zm00001d044327	LOG7	PSY	ABA_Biosynthesis
Zm00001d044442		CHYB	ABA_Biosynthesis
Zm00001d044443		CHYB	ABA_Biosynthesis
Zm00001d044906	opr2	ABCGs	ABA_Transportation
Zm00001d044908	opr1	ABCGs	ABA_Transportation
Zm00001d045046		ABCGs	ABA_Transportation
Zm00001d045112	arr2	ABCGs	ABA_Transportation
Zm00001d047017		PP2C	ABA_Signal_Transduction
Zm00001d047209		PP2C	ABA_Signal_Transduction
Zm00001d047743	fad7	PP2C	ABA_Signal_Transduction
Zm00001d047758	mpk1	PP2C	ABA_Signal_Transduction
Zm00001d048199	gras45	PP2C	ABA_Signal_Transduction
Zm00001d048263	zim4	PP2C	ABA_Signal_Transduction
Zm00001d049540	nactf77	CDPK	ABA_Signal_Transduction
Zm00001d050768	crr7	ABD1	ABA_Signal_Transduction
Zm00001d051863		ENT	CTK_Transportation

Zm00001d052530		HD-ZIP	CTK_Signal_Transduction
Zm00001d052653	rboh4	IPT	CTK_Biosynthesis
Zm00001d052725	bzip43	IPT	CTK_Biosynthesis
Zm00001d053436		ZOGT	CTK_Biosynthesis
Zm00001d002592	acs2	ACS	ET_Biosynthesis
Zm00001d017592	ereb16	EREBP	ET_Signal_Transduction
Zm00001d024843	acco31	ACO	ET_Biosynthesis
Zm00001d031796	ereb172	EREBP	ET_Signal_Transduction
Zm00001d033862	acs6	ACS	ET_Biosynthesis
Zm00001d047758	mpk1	MPK3	ET_Signal_Transduction
Zm00001d052229	ereb17	EREBP	ET_Signal_Transduction
Zm00001d003850		SBP	BR_Signal_Transduction
Zm00001d003852		SBP	BR_Signal_Transduction
Zm00001d005826	umc5a	BIS	BR_Signal_Transduction
Zm00001d005969		BIM1	BR_Signal_Transduction

Zm00001d006065		HBI1	BR_Signal_Transduction
Zm00001d006677	bzr7	BES1/BZR1	BR_Signal_Transduction
Zm00001d006947		castasteroneC-26hydroxylase	BR_Biosynthesis
Zm00001d006948		castasteroneC-26hydroxylase	BR_Biosynthesis
Zm00001d008307	nactf97	AIFs	BR_Signal_Transduction
Zm00001d008308	nactf97	AIFs	BR_Signal_Transduction
Zm00001d019139	smt1	SMT1	BR_Biosynthesis
Zm00001d020639	umc5b	BIS	BR_Signal_Transduction
Zm00001d028686		HERK1	BR_Signal_Transduction
Zm00001d035392		cycloartenolsynthase	BR_Biosynthesis
Zm00001d036112		SBI1	BR_Signal_Transduction
Zm00001d037544	cl12999_1	HERK1	BR_Signal_Transduction
Zm00001d039439	bzr2	BES1/BZR1	BR_Signal_Transduction
Zm00001d039642		castasterone23-O-glucosyltransferase	BR_Biosynthesis
Zm00001d039650		castasteroneC-26hydroxylase	BR_Biosynthesis
Zm00001d040318	nactf66	AIFs	BR_Signal_Transduction

Zm00001d046422	brc2	castasteroneC-26hydroxylase	BR_Biosynthesis
Zm00001d048345	bsk1	BSK1	BR_Signal_Transduction
Zm00001d051484	IDP8534	SBP	BR_Signal_Transduction
Zm00001d052651	xth6	TCH4	BR_Signal_Transduction
Zm00001d053775	sbp19	SBP	BR_Signal_Transduction
Zm00001d006900	umc1464	2-dehydro-3-deoxyphosphoheptonatealdolase	SA_Biosynthesis
Zm00001d011133	wrky68	WRKYs	SA_Signal_Transduction
Zm00001d011527	wrky42	WRKYs	SA_Signal_Transduction
Zm00001d012482	wrky115	WRKYs	SA_Signal_Transduction
Zm00001d013098	ao2	AAO	SA_Biosynthesis
Zm00001d015025	pco789	cinnamate:CoAligase	SA_Biosynthesis
Zm00001d015515	wrky48	WRKYs	SA_Signal_Transduction
Zm00001d020492	wrky53	WRKYs	SA_Signal_Transduction
Zm00001d034073	wrky3	WRKYs	SA_Signal_Transduction

Zm00001d036726	wrky114	WRKYs	SA_Signal_Transduction
Zm00001d037607	wrky125	WRKYs	SA_Signal_Transduction
Zm00001d039245	wrky93	WRKYs	SA_Signal_Transduction
Zm00001d043025	wrky43	WRKYs	SA_Signal_Transduction
Zm00001d043060	wrky81	WRKYs	SA_Signal_Transduction
Zm00001d043062	wrky4	WRKYs	SA_Signal_Transduction
Zm00001d053369	wrky8	WRKYs	SA_Signal_Transduction
Zm00001d003850		SPL	SL_Signal_Transduction
Zm00001d003852		SPL	SL_Signal_Transduction
Zm00001d021410	psy3	PSY	SLs_Biosynthesis
Zm00001d028094	si7762c1	D14	SL_Signal_Transduction
Zm00001d051484	IDP8534	SPL	SL_Signal_Transduction
Zm00001d053775	sbp19	SPL	SL_Signal_Transduction

.
